# Empowerment of Cancer Survivors Through Information Technology: An Integrative Review

**DOI:** 10.2196/jmir.4818

**Published:** 2015-11-27

**Authors:** Wim G Groen, Wilma Kuijpers, Hester SA Oldenburg, Michel WJM Wouters, Neil K Aaronson, Wim H van Harten

**Affiliations:** ^1^ The Netherlands Cancer Institute Division of Psychosocial Research and Epidemiology Amsterdam Netherlands; ^2^ The Netherlands Cancer Institute Division of Surgical Oncology Amsterdam Netherlands; ^3^ University of Twente Department of Health Technology and Services Research Enschede Netherlands

**Keywords:** review, neoplasms, Internet, technology, health education, chronic disease, power (psychology)

## Abstract

**Background:**

Patient empowerment may be an effective approach to strengthen the role of cancer survivors and to reduce the burden on health care. However, it is not well conceptualized, notably in oncology. Furthermore, it is unclear to what extent information technology (IT) services can contribute to empowerment of cancer survivors.

**Objective:**

We aim to define the conceptual components of patient empowerment of chronic disease patients, especially cancer survivors, and to explore the contribution of existing and new IT services to promote empowerment.

**Methods:**

Electronic databases were searched to identify theoretical and empirical articles regarding empowerment. We extracted and synthesized conceptual components of patient empowerment (ie, attributes, antecedents, and consequences) according to the integrated review methodology. We identified recent IT services for cancer survivors by examining systematic reviews and a proposed inventory of new services, and we related their features and effects to the identified components of empowerment.

**Results:**

Based on 26 articles, we identified five main attributes of patient empowerment: (1) being autonomous and respected, (2) having knowledge, (3) having psychosocial and behavioral skills, (4) perceiving support from community, family, and friends, and (5) perceiving oneself to be useful. The latter two were specific for the cancer setting. Systematic reviews of IT services and our additional inventory helped us identify five main categories: (1) educational services, including electronic survivorship care plan services, (2) patient-to-patient services, (3) electronic patient-reported outcome (ePRO) services, (4) multicomponent services, and (5) portal services. Potential impact on empowerment included knowledge enhancement and, to a lesser extent, enhancing autonomy and skills. Newly developed services offer promising and exciting opportunities to empower cancer survivors, for instance, by providing tailored advice for supportive or follow-up care based on patients' input.

**Conclusions:**

We identified five main components of empowerment and showed that IT services may especially contribute to empowerment by providing knowledge. The components of empowerment could be used to develop IT services for cancer survivors. It is important to take into account patients’ needs, follow up on these needs, and create a service that is attractive and easy to use.

## Introduction

A popular approach to improve the involvement of patients in their care is to provide them with information on how to make a decision about medical treatment (ie, shared decision making) [1]. However, more aspects are important when fully engaging patients regarding their health. A concept that may be particularly relevant in this regard is patient empowerment, which is generally viewed as a multilevel construct with manifestations at the community, group, or individual level. Despite its popularity, patient empowerment has no generally accepted definition or conceptualization and it is a rather complex and multifaceted construct. This is illustrated by the differences in various questionnaires that have been developed to measure aspects of empowerment [2]. Nevertheless, there is convincing evidence for the effects of improving patient empowerment on health outcomes. Meta-analyses show that self-management interventions improve glycated hemoglobin levels (a marker of glycemic control), self-efficacy, and empowerment levels in patients with chronic metabolic diseases [3]. Moreover, these interventions reduce the number of readmissions due to heart failure [4] and emergency department visits due to asthma [4].

Because of better screening, detection, and treatment, the number of cancer survivors is growing rapidly. We will use the term cancer survivor as the National Coalition of Cancer Survivorship defines cancer survivorship: “from the time of diagnosis and through the balance of life.” In 2008, 28.8 million people worldwide had survived cancer for at least 5 years [5]. Cancer and its treatment result in a wide range of physical and psychological challenges, some of which may even appear years later [6]. The current models of survivorship care are likely to lead to rapidly increasing and not sustainable use of health care [7]. Some claim that a stronger role of the patient might be helpful to control costs. New models are emerging that emphasize the importance of supporting patients to engage in self-management activities and to be able to make informed choices about the type of support they need. The challenge is to provide this in a cost-effective way that is either equally or more effective than traditional models of survivorship care [8]. It seems imperative that cancer survivors need to become more effective coactors in their health care. Existing theories and models of chronic disease management might be relevant to cancer survivors as well, but have not been tested rigorously [9,10]. In this review, we therefore focused on the *higher order* concept of patient empowerment.

Empowering interventions providing face-to-face support to patients require substantial resources and effort. A promising approach is the use of information technology (IT), which enables the provision of easily accessible, up-to-date, tailored information and automated feedback to patients. Many *empowering* Web-based interventions have been developed in the field of chronic diseases (eg, diabetes, heart failure, and chronic obstructive pulmonary disease), but relatively few seem to have been developed for, and rigorously tested in, cancer survivors [11].

The objective of this study is to identify conceptual components of patient empowerment in chronic patients and cancer survivors and to explore the contribution of existing and new IT services to promote their empowerment. This can guide the development of innovative and sustainable eHealth services that may improve empowerment in cancer survivorship care.

## Methods

### Overview

We conducted an integrative literature review using the methodology as proposed by Whittemore and Knafl [12] to conceptualize the construct of empowerment. An integrative review summarizes past empirical and theoretical literature to provide a more comprehensive understanding of a phenomenon or health care problem [12]. Accordingly, five steps were undertaken: (1) problem identification (already stated in the introduction), (2) literature search, (3) data evaluation, (4) data analysis, and (5) presentation. In addition, we searched for IT services that could support cancer survivors with regard to patient empowerment. The Preferred Reporting Items for Systematic Reviews and Meta-Analyses (PRISMA) statement was checked for relevant items that would aid the reporting of this review [13].

### Integrative Literature Review

#### Literature Search and Data Evaluation

We performed a literature search in PubMed (Medline), Scopus, and PsycINFO from January 1990 up to April 2014 to obtain definitions of patient empowerment in general, and specifically in cancer survivors. Search terms included “conceptual,” “theory,” and “cancer” either alone or combined, and always in combination with “patient empowerment.” The search query for PubMed is presented in Multimedia Appendix 1. We selected publications for full-text review based on screening of titles and abstracts. Articles were selected for inclusion if they met the following criteria: (1) were written in English, (2) were published in a peer-reviewed journal, and (3) were a specific type of article. Specific article types included in the study were as follows: (1) articles providing a conceptual or theoretical description of patient empowerment, (2) articles providing empirical qualitative data on the concept of patient empowerment in chronic patients or cancer survivors, (3) articles describing the development of a questionnaire that aims to measure aspects of patient empowerment, both generic and disease specific, and (4) quantitative articles with empowerment as an outcome, which also extensively discuss the conceptual definition of patient empowerment in chronic patients or cancer survivors. Reference lists of selected full texts were screened for additional relevant papers. Two researchers (WG, WK) reviewed all articles for inclusion, and in case of disagreement a third researcher (WvH) was consulted for a definitive decision.

#### Data Analysis and Presentation

A predefined data sheet was used for data extraction. Data were extracted on study characteristics (ie, first author, year of publication, type of research, number of participants involved, and type of disease), defining attributes (ie, characteristics), antecedents (ie, events or circumstances that precede a concept), and consequences (ie, phenomena that follow an occurrence of the concept) of empowerment [14]. In order to identify the main attributes, we coded qualifying text elements and these were integrated into common descriptions. A final check of the primary data sources was performed to verify the conceptualization [12]. Findings of the studies on cancer, specifically, were reviewed separately and compared to the findings of the literature on chronic diseases. All aspects of the procedure were verified by a second researcher (WK) to reduce potential bias. Data are presented narratively.

### Identification of Existing Information Technology Services and Their Potential to Support Patient Empowerment in Cancer Survivors

We searched the Medline (PubMed) database for papers in English that were published between January 2010 and January 2015. We used medical subject headings and free-text terms. A combination of any of the terms "cancer patient," "cancer survivor," "cancer survivorship," and “cancer” was combined with any combination of the terms “information and communication technology” (ICT), "ICT," "Web-based," and "Internet." The search query for PubMed is presented in Multimedia Appendix 1. Because there have been several recent, systematic reviews on this topic, we decided not to do a search for primary articles. One author (WG) checked titles and abstracts to determine if articles were sufficiently relevant to retrieve the full-text article. Reference lists were also searched for additional relevant papers. Full texts were screened by two reviewers (WG, WK) on the following criteria: (1) the paper described a literature review with a systematic and explicit search strategy, (2) the scope of the review concerned supportive IT services, (3) the services focused on adult cancer survivors, and (4) the paper contained information on the features and effects of the reported IT services or, when these were not reported, obtained this from primary articles.

Apart from the services described in the systematic reviews, there were also emerging services that were in an early phase of development and had not yet been rigorously tested. We therefore additionally searched Google and PubMed up to April 2014. To our knowledge, no formal guidelines exist for surveying eHealth systems and applications. We purposely selected three groups of these IT services that may be particularly relevant for cancer survivors (but may be designed for other diseases as well), namely: (1) patient portals, (2) electronic patient-reported outcome (ePRO) systems, and (3) IT services related to survivorship care plans for cancer patients. Major keywords used, either alone or in combination, were as follows: "patient portal(s)," "patient empowerment," "electronic medical record," "patient reported outcomes," "survivorship care plan," "cancer," and "information and communication technology." References in reports and articles were checked for citations leading to other possible relevant IT services (ie, snowballing method). Suggestions from members of our project group were added to this inventory as well. Our inclusion criteria were as follows: (1) we included portal services that at least provided insight into the patient's medical record, (2) with regard to ePRO systems, we included systems that enable people with cancer to complete symptoms and/or quality-of-life questionnaires by computer, either at home or at the clinic, (3) for the survivorship care support services, we included those that were aimed at cancer survivors and provided an electronic survivorship care plan (SCP) or generated one from a digital registry, and (4) all IT services had to be in use and not only available as a test or beta version.

From the identified IT services, key features and user interaction aspects were collected from websites, published manuscripts, or by demos of the service when possible. Lastly, convenience sample-based site visits were made to developers of the OncoKompas system (Free University Medical Center, Amsterdam, Netherlands), the ChipSoft patient portal (University Medical Center [UMC] Utrecht, Netherlands), the digital in vitro fertilization (IVF) clinic (Radboud UMC, Nijmegen, Netherlands), and the electronic patient-reported outcome system of UMC Leiden, Netherlands. The developers of Care Companion (Sanofi Aventis, Gouda, Netherlands) visited us to demonstrate their application. Demo software of the Computer-based Health Evaluation System (CHES) for ePRO (Innsbruck, Austria) was obtained for review. References to the services can be found in Multimedia Appendix 2 [15-25].

The possible contribution of identified IT services to attributes of patient empowerment was determined by relating the reviewed features and effects to the attributes of empowerment as identified by the integrative review. Results are presented qualitatively and several exemplary IT services are described in detail.

## Results

### Integrative Literature Review

#### Overview

**Figure 1:**
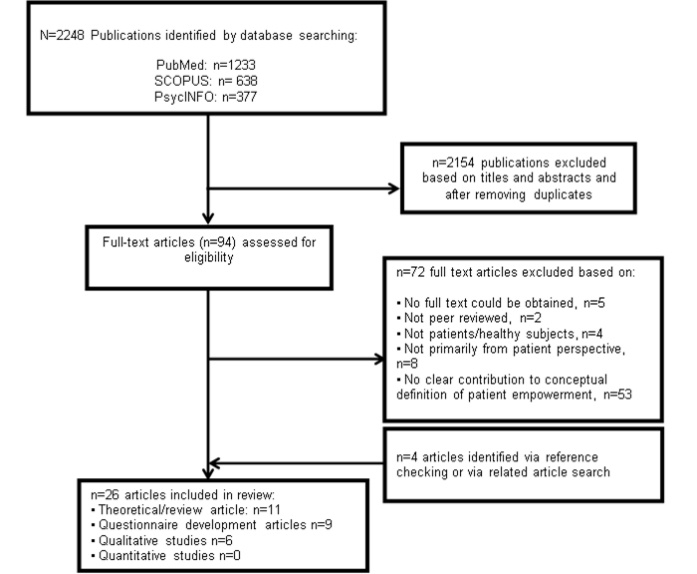
Flowchart of selection of articles according to PRISMA.

**Figure 2:**
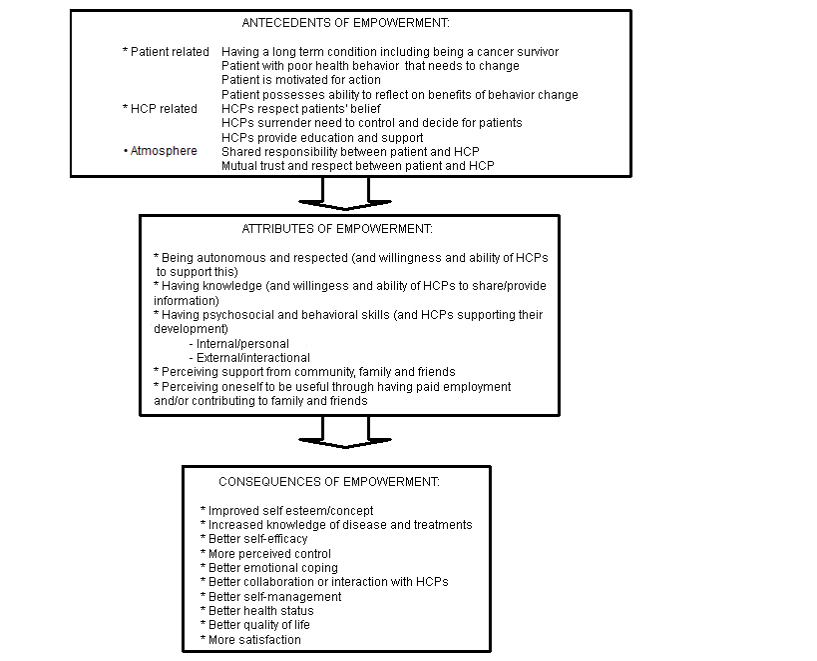
Conceptual components of empowerment in chronic patients, including cancer survivors. HCP: Health care provider. A detailed overview of attributes is provided in Tables 1 and 2.

The initial search resulted in 2248 hits. After screening titles and abstracts, 94 papers were selected and read in full text. A total of 22 papers met our inclusion criteria and four articles were added by checking reference lists. A total of 11 reviews were included [26-36]. Nine manuscripts described the development and/or psychometric evaluation of a questionnaire on patient empowerment [37-45] and six qualitative studies described patient empowerment of chronic patients [46-51]. Figure 1 shows the literature search and selection procedure. Antecedents, attributes, and consequences of patient empowerment of chronic patients and cancer survivors are presented in Figure 2 and are described in more detail below. References to the most representative manuscripts are provided.

#### Antecedents

Antecedents that appeared from the literature were to a large extent based on the excellent review of Holmström et al [32]. Antecedents are events or circumstances that precede a concept [14]. An antecedent may contribute to the occurrence of the concept, it may be associated with its occurrence, or it may need to be present for the concept to occur. Several antecedents of patient empowerment are related to patients themselves. Having a long-term condition [35,40] is a first antecedent of empowerment. Further antecedents on the patient side are poor health behaviors that need to be changed, the presence of motivation for action toward desired goals(s), and the ability to self-reflect regarding benefits of behavior change [32].

There are several antecedents of patient empowerment that relate to health care providers (HCPs) and their approach to patients. When HCPs respect patients’ beliefs and surrender their need to control and decide for patients, they create an atmosphere of mutual trust and respect [28,29,31] and shared responsibility [32], which facilitates patient empowerment. Finally, an antecedent to patient empowerment is HCPs’ willingness to provide educational support to patients [28,32].

#### Attributes

Three main attributes emerged from the literature review concerning chronic patients.

##### Being Autonomous and Respected (and Willingness of Health Care Providers to Support This)

Patients must have the opportunity to make their own decisions and choose their own health or life goals [26,28,30,34]. This attribute depends, in part, on external factors. For example, HCPs need to adjust their position of power to a level that provokes equal participation of the patient, and they need to act as a coach to work toward negotiated health goals [26,29-31,35,36]. Being respected also requires that HCPs share knowledge and resources in such a way that patients feel fully recognized [29,35].

##### Having Knowledge (and Willingness and Ability of Health Care Providers to Share/Provide This)

Having knowledge about one’s health situation is another attribute of empowerment. It refers to having knowledge about one’s disease [45,50,51], about oneself [30,34], and about available supporting resources [28,34]. Having more knowledge is expected to enable "better informed decision making," for example, about treatments or lifestyle changes [31,50,51].

##### Having Psychosocial and Behavioral Skills (and Health Care Providers Supporting Their Development)

Nearly every article that we reviewed described having skills to positively influence one’s situation as an attribute of patient empowerment. These skills could be subdivided into those related to internal thought processes (ie, internal/personal) and those more behaviorally and externally oriented (ie, external/interactional). Internal/personal skills are, in general, referred to as personal psychological strengths, such as having a sense of self-efficacy, self-esteem, optimism, and personal competence [41,49], as well as the ability to accept and cope with living with a chronic disease [40,45,49]. These skills are also related to building the capacity to identify one’s needs, psychosocial problems, goal setting, and problem solving [36]. It also refers to the skill of patients to increase and maintain motivation to pursue their health goals [36].

External/interactional skills are needed to positively influence one’s current situation by one’s own behavior and/or by interaction with others. These skills are generally related to the effectiveness of patients in managing their disease through preventive self-management [30,34,38,39,42,45,50] and effective, collaborative interaction with HCPs, such as negotiating and asking for clarification [36,38]. Furthermore, being able to obtain emotional and practical support from family and friends is important [37,38,40,41,43,47,48]. Using or developing these skills may be a challenge for patients who have, or have had, a life-threatening disease such as cancer. Nevertheless, developing or reinforcing these skills is the hallmark of empowering interventions [36].

#### Consequences

A diversity of consequences of empowerment was reported in the reviewed literature. Empowerment is associated with increased self-esteem and a better self-concept [27,28], increased knowledge about disease and treatments [32,35,36,40,46], more self-efficacy regarding disease and treatment-related behaviors [27,29,32,36,47], and more perceived control [27,28,36,40]. It is also associated with better interaction or collaboration with HCPs [32,42], better disease self-management [32,35,36,42], and improved emotional coping [32,36,37,39]. In more general terms, empowerment is related to higher levels of satisfaction [27,29,32,36], better health status [29,32,36,39,42], and better quality of life [27,29,36,47].

**Table 1:** Identified attributes of patient empowerment and the possible contributing role of reviewed educational and patient-to-patient IT^a^services.

Attributes of empowerment			Possible contributing role of reviewed IT services	
			Educational services	Patient-to-patient services
**Being autonomous and respected (and willingness and ability of HCPs** ^b^ **to support this)**				
	Patients make their own decisions and choose their own health or life goals		o^c^	+^d,e^
	There is an atmosphere of mutual trust		o	o
	HCPs bring their power to a level that provokes equal participation of the patient and act as coaches to work toward negotiated health goals		o	o
	HCPs share knowledge and resources in such a way that patients feel fully recognized		++^f,g^	o
**Having knowledge (and willingness and ability of HCPs to share/provide information)**				
	Having knowledge about one’s disease and treatments and about oneself		++^h^	+^i^
	Having knowledge about available supporting resources		++^h^	+^i^
**Having psychosocial and behavioral skills (and HCPs supporting their development)**				
	**Internal/personal**			
		Having self-efficacy, self-esteem, optimism, and personal competence	o	o
		Ability to accept diagnosis and cope with emotions (eg, anxiety or depression)	+^j^	o
		Capacity to identify one’s needs and psychosocial problems and set goals to improve self-selected goals	o	o
		Increase and maintain motivation to pursue health goals	o	o
	**External/interactional**			
		Effectiveness of patients in managing their disease through preventive self-management	+^k^	o
		Effective collaborative interaction with HCPs, such as negotiating, asking for clarification, etc	o	o
		Being able to obtain emotional and practical support from family and friends	+^k^	o
Perceiving support from community, family, and friends			o	+^l^
Perceiving oneself to be useful through having paid employment and/or by contributing to family and friends			o	o

^a^IT: information technology.

^b^HCP: health care provider.

^c^o: no positive contribution to empowerment.

^d^+: weak positive contribution to empowerment.

^e^Patients can ask questions regarding their most relevant issues in online communities.

^f^++: strong positive contribution to empowerment.

^g^Knowledge and resources are shared between HCPs and patients.

^h^Providing Information about, for example, diagnosis, treatments, side effects, late effects, follow-up scheme, and healthy lifestyle, and information on, or links to, supporting resources.

^i^Information could be obtained through online communities, however quality may be limited.

^j^Training programs could enhance coping with emotions.

^k^Providing information about diagnosis, treatments, side effects, late effects, follow-up scheme, and healthy lifestyle, and information on, or links to, supporting resources. Provision of skill-building programs to effectively obtain social support.

^l^Patients could perceive more support from community (ie, fellow patients).

**Table 2:** Identified attributes of patient empowerment and the possible contributing role of reviewed ePRO^a^services and patient portals.

Attributes of empowerment			Possible contributing role of reviewed IT^b^services	
			ePRO services	Patient portals
**Being autonomous and respected (and willingness and ability of HCPs to support this)**				
	Patients make their own decisions and choose their own health or life goals		+^c,d^	o^e^
	There is an atmosphere of mutual trust		o	o
	HCPs bring their power to a level that provokes equal participation of the patient and act as coaches to work toward negotiated health goals		+^d^	o
	HCPs share knowledge and resources in such a way that patients feel fully recognized		o	++^f.g^
**Having knowledge (and willingness and ability of HCPs to share/provide information)**				
	Having knowledge about one’s disease and treatments and about oneself		+^h^	++^i^
	Having knowledge about available supporting resources		o	++^i^
**Having psychosocial and behavioral skills (and HCPs supporting their development)**				
	**Internal/personal**			
		Having self-efficacy, self-esteem, optimism, and personal competence	o	o
		Ability to accept diagnosis and cope with emotions (eg, anxiety or depression)	o	o
		Capacity to identify one’s needs and psychosocial problems and set goals to improve self-selected goals	+^j^	o
		Increase and maintain motivation to pursue health goals	+^j^	o
	**External/interactional**			
		Effectiveness of patients in managing their disease through preventive self-management	o	o
		Effective, collaborative interaction with HCPs, such as negotiating, asking for clarification, etc	++^k^	+^l^
		Being able to obtain emotional and practical support from family and friends	o	o
Perceiving support from community, family, and friends			o	o
Perceiving oneself to be useful through having paid employment and/or by contributing to family and friends			o	o

^a^ePRO: electronic patient-reported outcome.

^b^IT: information technology.

^c^+: weak positive contribution to empowerment.

^d^ePROs give patients the opportunity to identify personally relevant issues and health goals.

^e^o: no positive contribution to empowerment.

^f^++: strong positive contribution to empowerment.

^g^Medical knowledge in the electronic medical record (EMR) is shared between HCPs and patients.

^h^Providing knowledge of personal symptoms and physical and psychosocial functioning by providing graphic overview of symptom and quality-of-life scores.

^i^Providing information about diagnosis, test results, treatments, etc, by providing access to parts of the EMR during and after treatment. Providing knowledge about available supporting resources through tailored patient educational material.

^j^Identification of personal needs and psychosocial problems by providing graphic overview of symptoms and quality-of-life scores, with or without reference values.

^k^When ePROs are fed back to patients with coaching statements, it may improve the effectiveness of the encounters with health professionals.

^l^E-consultations may enhance patient-provider interaction.

#### Cancer-Specific Findings

Six papers were specifically aimed at cancer patients: three methodological papers describing the development and psychometric testing of a questionnaire designed to measure empowerment [37,38,41], and three very small-scale qualitative studies of which one—Bulsara et al [47]—was also included in a questionnaire development paper [46-48].

Taken together, the cancer-specific papers reflected many aspects already identified from literature on patients with a chronic disease. No cancer-specific antecedents or consequences could be identified from these studies. Most attributes were related to having knowledge and having psychosocial and behavioral skills. Cancer-specific attributes that could be added to the three already described above are as follows:

1. Perceiving support from community, family, and friends [37,41,48]. This refers to perceived support from people close to the patient and feelings of acceptance and support from the social community. It is about the availability of support, which makes it slightly different from the earlier identified skill of seeking support from these sources.

2. Perceiving oneself to be useful through having paid employment and/or by contributing to family and friends [37]. This attribute refers to the patient's sense of self-worth through having a job or through having the feeling that one is contributing to family or friends, or that they may rely on him/her.

Also, one specific subattribute could be added to the attribute, *having skills*: being able to accept the diagnosis [37,47] and coping with emotions (eg, anxiety or depression) related to the disease [38].

### Identification of Existing Information Technology Services and Their Potential to Support Patient Empowerment in Cancer Survivors

#### Identified Information Technology Services, Their Features, and Their Effects

The initial search identified 216 potentially relevant reviews. We selected 26 reviews for full-text screening and four met all of our inclusion criteria [52-55]. Two reviews were concerned with cancer in general [52,53], one was focused on breast cancer [54], and one on prostate cancer [55]. None of the included reviews performed a meta-analysis. The four reviews included 46 unique IT services (described in 74 studies). The additional search and expert input yielded additional information on five patient portal services, six ePRO services, and three electronic survivorship care plan support systems [15-25] (see Multimedia Appendix 2 for an overview). IT services could be divided into five categories: (1) educational services (including electronic survivorship care plan services), (2) patient-to-patient services, (3) ePRO services, (4) multicomponent services (which combine two or more of the former), and (5) patient portal services.

Below, we report on the main features of the services per category, including several examples and a summary of the evidence regarding the effectiveness or hypothesized benefits in supporting the attributes of patient empowerment, as identified in the previous step. For convenience, the possible contributions to attributes of empowerment are indicated as follows: being autonomous and respected (AR); having knowledge (HK); having skills (SK); and perceived support from community, family, and friends (PS). We felt that IT could not/hardly contribute to the attribute, *Perceiving oneself to be useful through having paid employment and/or by contributing to family and friends*; therefore, this attribute is not represented here. An overview is presented in Tables 1 and 2. Because the features of multicomponent services were very diverse, these are not presented in the tables.

#### Educational Services

Educational services often are interactive systems provided via the Internet or offline via computer or CD-ROM. Many provide disease- and treatment-related information to improve knowledge of survivors (HK). Some are specifically aimed at improving decision making (SK), for example, regarding surgical treatment for breast or prostate cancer. An example is the Interactive Digital Education Aid (IDEA) intervention [56]: an interactive software program that includes high-quality, three-dimensional animated graphics, patient testimonials, before-and-after photographs, and video explanations from clinical specialists. It is designed to answer general questions about breast reconstruction and to provide detailed explanations of the various techniques, including advantages and disadvantages (HK). The intervention has been shown to increase the knowledge (HK) and satisfaction with treatment choice, compared to a control group.

Other services contain educational programs about symptoms and how to cope with them (HK and SK). For example, the Sleep Healthy Using The Internet (SHUTi) program is based on a well-validated, face-to-face cognitive behavioral therapy (CBT) program and includes six interactive modules [57]. It covers aspects of behavior, stimulus control, education, and problem prevention (SK). SHUTi provides a high degree of individual tailoring and feedback. Automated emails are sent throughout the program to inform the users about next steps, as well as to encourage adherence. A small-scale, two-arm randomized controlled trial (RCT) (n=14 per group) showed that the intervention significantly improved overall insomnia severity, sleep efficiency, sleep onset latency, soundness of sleep, restored feeling upon awakening, and general fatigue.

The electronic survivorship care plan support is based on the recommendation of the Institute of Medicine that survivors should be provided with a personalized structured overview of one’s disease, treatments, and possible short- and long-term side effects (HK). Furthermore, it may include information on available resources, recommended follow-up visits, and on healthy lifestyle (AR and HK). Several electronic SCP initiatives exist. The LIVESTRONG Care Plan provides an online SCP based on the data that patients provide themselves [23]. Patients can then view and/or print their SCP. Other systems such as ROGY Care (Registrationsystem Oncological GYnecology) [25] and SCP Builder [24] enable health professionals to compose an SCP for survivors based on tumor registry data, manual input, or both. The electronic SCP initiatives are hypothesized to contribute to patient empowerment by increasing autonomy through HCPs sharing knowledge about available resources. Furthermore, it may enhance patients’ knowledge of their current and future situation. With regard to skill development, SCPs appear to offer limited benefit. Patients might better prevent side effects and late effects by adopting a healthier lifestyle.

Beneficial effects of educational services related to patient empowerment include increased levels of knowledge, skill development through better decision making, increased levels of satisfaction, and, to a lesser extent, a better quality of life. However, the number of high-quality studies supporting those claims remains limited [52-55].

#### Patient-to-Patient Services

Patient-to-patient services consist of online support groups or bulletin boards in which patients can exchange experiences with fellow patients and ask about their most bothersome issues (AR, HK, and PS). These services are quite unstructured and the quality of feedback may be limited because fellow patients may have different treatments and may lack proper medical knowledge. An example is the Internet peer support offered to a large group of cancer survivors as described by Hoybye et al [58]. The intervention contained a self-guided space for communication, including an Internet discussion forum, a live chat room, and a personal message system. Groups would form around a shared cancer diagnosis or a particular shared concern in relation to the experience of cancer (HK and PS). No therapeutic content or information services were offered within the groups. The intervention did not result in statistically significant improvements of self-reported mood disturbance, adjustment to cancer, or self-rated health.

Patient-to-patient services may contribute to empowerment through increased autonomy, because patients can ask questions about their most bothersome issues, and through increased knowledge about one’s health/disease. Furthermore, it may improve perceived support from the community, in this case fellow patients. To date, however, there are few studies available to make firm claims on effectiveness related to patient empowerment. Results of controlled studies range from positive effects on depression, cancer-related trauma, and perceived stress [59] to no effects and even negative effects on psychosocial distress and quality of life [60].

#### Electronic Patient-Reported Outcome Services

Several national and international services have been developed whereby cancer survivors can complete validated questionnaires about symptoms and/or quality-of-life issues at home or in the waiting room of the clinic. All identified systems possess the ability to graphically show questionnaire data to clinicians, and some have the feature of adding norm values derived from healthy individuals, such as the CHES [20]. Few services provide graphic feedback on scores or outcomes to patients themselves. Most of these systems are not directly integrated with the electronic medical record (EMR), but with freestanding database systems that can be linked via a standardized Health Level Seven (HL7) protocol. The Electronic Self-Report Assessment for Cancer (ESRA-C) system [61] enables patients to score their symptoms and quality of life and get tips for communicating about their most bothersome issues with HCPs (SK). Furthermore, patients can review all aspects of their symptoms and quality of life in charts with cutoff values (HK) and may be better able to identify their personal needs and psychosocial issues (SK). A promising new approach is the OncoKompas that provides survivors with an overview of one’s health status in terms of a profile indicating whether the patient is either “on track” or “off track” [22]. This profile is based on a set of validated questionnaires and uses an internal algorithm and cutoff values. It also provides advice on how to address problems (HK) using a stepped-care algorithm, starting with self-help options (SK) and gradually progressing to professional help. It has not been tested in a controlled study. Another promising system is the electronic patient self-assessment and management (SAM) framework which provides tailored information and graphical feedback to prostate cancer patients postsurgery based on their input [21].

ePRO systems are hypothesized to contribute to patient empowerment by enabling survivors to identify their most relevant issues (ie, increased autonomy) and by enhancing their knowledge of their current health status and patterns of change in their health over time. Having such information may also increase the effectiveness of the encounters of patients with their HCPs; both patients and their HCPs may be motivated to talk about the patients' most bothersome issues. Two RCTs of the ESRA-C have demonstrated its effectiveness in terms of the number of bothersome issues discussed (HK and SK) and a small improvement in symptom distress (SK) [61,62].

#### Multicomponent Services

Many services contain a mix of the aforementioned features. Most services combine educational support with patient-to-patient support as described above. One such service is the Comprehensive Health Enhancement Support System (CHESS) [63]. This service provides access to many online services, including information services such as breast cancer information, personal stories of fellow patients, a resource guide, discussion groups, ask an expert, live chats, and coaching services [63].

Because this category of services is quite broad, the evidence regarding effects on enhancing empowerment is also variable. There is some evidence that these services contribute to enhanced knowledge and skills, mostly from studies of CHESS. However, other services also show improved knowledge [64] and coping [65] skill as a result of educational and patient-to-patient support.

#### Patient Portal Services

Most patient portals offer patients access to their EMR (AR and HK) and provide additional services like tailored patient education (HK), questionnaire administration, posing questions electronically to clinicians (ie, e-consultation) (SK), making notes/keeping a diary for oneself (HK), ordering medication, and appointment keeping (SK). Most portals are directly integrated with the EMR, although some are separate applications that connect to the EMR and transfer data to a freestanding Web-based portal [6]. In the Netherlands, portals are secured via a two-way authorization procedure, meaning that patients have to provide a personal username and password, and consequently have to enter an additional code that is sent to their mobile phone.

No controlled studies have investigated the effect of patient portals on improving aspects of empowerment in cancer survivors, but they may contribute to patient empowerment in several ways. First, by accessing their EMR, patients have the same clinical information available as their clinicians, thus potentially enhancing perceptions of being respected and of autonomy. Second, portals may enhance patients’ knowledge of their disease and treatments, either via access to their EMR or via tailored patient education. To date, there appears to be limited contribution of patient portals to skill development. E-consultation might enhance the effectiveness of the interaction of patients with their HCPs.

## Discussion

### Overview

By using the integrative review methodology, we were able to identify conceptual components of patient empowerment of chronic patients including cancer survivors. We illustrated the various ways in which selected IT services may contribute to patient empowerment of cancer survivors. This is a first attempt to link empowerment theory to existing IT services for cancer survivors.

### Patient Empowerment of Cancer Survivors

We identified five main attributes related to patient empowerment. The first is *being autonomous and respected (and willingness and ability of HCPs to support this)*. For this attribute, a patient-centered approach seems imperative. Patients need to be regarded as a valuable source of information, and the goals that are set should be derived from the patients themselves. These goals could, but do not have to, correspond with the ones based on the health provider’s values. When external factors are neglected (eg, how clinicians approach patients), it may be difficult to facilitate empowerment in patients. We cannot expect patients to take an active role when that is not supported by the health care environment. It is known that patients in general depend highly on their oncologists, which consequently leads to high levels of trust. This high dependency and need for trust might result in a more obedient attitude and reduced autonomy of patients [66].

The second attribute is *having knowledge (and willingness and ability of HCPs to share/provide this)*. For this attribute, we have to realize that some patients may not want to know all the details of their disease and treatments. For example, it is known that people can have different coping styles with regard to information. There are *monitors*—those who attend to threatening information—and *blunters*—those who avoid it. In general, monitors are more physiologically, behaviorally, and subjectively aroused than blunters, and these differences occur primarily under conditions of threat of the sort when cancer risk or diagnosis is at issue [67]. Patients with a blunting coping style may be unable to process the relevant information necessary to make informed decisions regarding treatment or self-care. It is thus important to match the amount of information to patients' coping styles to reduce their levels of stress, because telling patients either more or less than they want to know about a stressor will make it more stressful [67].

The third identified attribute is *having psychosocial and behavioral skills (and HCPs supporting their development)*. In the literature, many empowering interventions have been described to enhance survivors’ skills. For example, one could think of a physical activity intervention with motivational interviewing technique [68] to enhance exercise behavior, or cognitive behavioral therapy to restructure dysfunctional ways of thinking to alleviate symptom burden (eg, CBT for climacteric symptoms [69] or for cancer-related fatigue [70]). Physical activity may be a particularly promising intervention for patient empowerment because, in addition to its direct beneficial effects on physical and psychosocial outcomes in cancer [71], it may also function as a gateway behavior, such that improvements in physical activity behavior positively influence other health behaviors, like healthy eating [72]. It may therefore be particularly useful to emphasize physical activity promotion in cancer survivorship.

The fourth and fifth attributes were derived from cancer-specific studies. *Perceiving support from community, family, and friends* was highlighted by several papers and delineates the importance of patients not only having skills to obtain support, but also the a priori existence of such sources of support which are strong enough to be perceived as supporting by the patient. This attribute seems to very difficult to facilitate with the use of IT. *Perceiving oneself to be useful through having paid employment and/or by contributing to family and friends* highlights the importance of maintaining a sense of self-worth and the importance of returning to work. Return-to-work programs specifically aimed at cancer survivors exist but are still relatively rare [73]. These cancer-specific attributes are highly relevant but appear not to be very actionable by IT services.

Empowerment appears to be a relatively new concept in its application to cancer survivors. This may be due to a recent paradigm shift; many cancer types are no longer regarded as a deadly disease, but as a disease with a chronic nature, requiring ongoing support and care. This is highlighted by the fact that existing theories and models of chronic disease management, such as the Chronic Care Model (CCM) and the Chronic Disease Self-Management Program have recently been introduced to cancer survivorship [9,10]. Not surprisingly, much overlap exists between these models and our identified attributes of empowerment. Interestingly, researchers have recently related the CCM to ICT [74,75], and alterations to the CCM have been proposed to make it more appropriate for eHealth use [75]. These existing models may also be considered for future IT services for cancer survivors.

Recently, others have attempted to conceptualize patient empowerment by systematically reviewing questionnaires purporting to capture patient empowerment [76] and by using a mixed-methods approach combining literature review and focus groups [77]. Although these studies have taken a slightly different approach than ours, the findings show great overlap, which strengthens the confidence in our findings. We found that the attributes of empowerment do not differ much for cancer survivors compared to patients with a chronic disease, however further in-depth research is needed, especially on empowerment in dealing with symptom-specific aspects and with cancer worries.

### Using Information Technology to Support Patient Empowerment in Cancer Survivors

#### Overview

For patients with a chronic disease we previously found that there are many IT services available that are aiming to increase aspects of empowerment through Web-based interventions [11]. Positive effects regarding empowerment were found, mainly related to self-care behavior and measures of self-efficacy [11]. In this study, we additionally found IT services that could contribute to the empowerment of cancer survivors, specifically. When we related these services to the earlier identified attributes of empowerment it showed that they contribute to attributes of empowerment differently. Mainly, services were found to contribute to enhancing knowledge of patients regarding their situation, but the evidence for enhancing skills was limited. The contribution to the attribute of *being autonomous and respected* may be present in some tailored services, however, it is hardly ever a specific study end point, which makes it hard to substantiate it. For the different types of IT services, we will now discuss points of attention with regard to empowerment of cancer survivors.

#### Educational Services

Educational services may greatly contribute to patient empowerment by providing patients with knowledge and skills. The approaches taken and the use of IT differs greatly, ranging from interactive decision tools to services that interactively deliver cognitive behavioral therapy to electronically provided survivorship care plans. Although appealing, the empowering potential of the latter service, either generated online or printed from an electronic tumor registry, seems to be mainly restricted to knowledge provision. To date, there is very little evidence of the effectiveness of providing patients with a survivorship care plan on aspects of patient empowerment [78]. From focus groups, we know that survivors anticipate that they would benefit from SCPs because they provide information about side effects that could occur in the long term and advice for a healthy lifestyle. They also value having an overview of diagnosis- and treatment-related information [79]. However, when focusing on the potential to empower cancer survivors, providing SCPs may be too passive an approach when it comes to enhancing skill levels. It is expected that when e-interventions are offered based on the input of patients (eg, as is done by the OncoKompas), cancer survivors will gain skills related to their problem areas. This hypothesis, however, needs to be tested in high-quality controlled trials. The use of SCPs could be valuable to facilitate information transfer from oncology centers to primary care and between several primary care physicians, especially in countries where the information transfer between these care providers is suboptimal.

#### Patient-to-Patient Services

Patient-to-patient services seemed to offer little benefit in terms of empowerment and some negative effects have been reported as well. In focus group sessions, we found that some survivors would find such a service useful, mainly for practical tips such as where to find good wigs; however, in general, they did not endorse such a feature. Many doubted the quality of the information due to lack of moderation by an HCP, and they were also reluctant in sharing their emotions via this medium [79]. On the other hand, new peer-to-peer services, such as *PatientsLikeMe,* seem to grow in popularity, indicating there may be value in it for patients that has not been studied and recognized yet.

#### Electronic Patient-Reported Outcome Services

A recent review shows that routine use of patient-reported outcomes (PROs) in clinical practice leads to better communication between patient and physician [80], and this benefit may also be expected from ePRO systems. These systems have the opportunity to immediately provide relevant, Web-based interventions to alleviate symptoms or improve coping ability for problems indicated on PROs. Of the reviewed IT services, these seem to be particularly promising as will be outlined in the following section.

#### Multicomponent Services

It remains unclear what positive effects are related to what mix of components, but a study of Baker et al [63] comparing different versions of the CHESS—information only versus information and support services versus information, support, and coaching services—showed that emotional coping was only enhanced in the first two conditions, contrary to their expectations. The authors stated that the full CHESS version may be too complex for survivors and may reduce its effectiveness. In line with this finding, the WebChoice service [81] failed to show major benefits, despite the fact that it contained educational, patient-to-patient, and ePRO services.

#### Patient Portals

Most portals that we reviewed are likely to make some contribution to patient empowerment. A recent systematic review questions the effectiveness of patient portals, as the data to date provide limited evidence for improved health outcomes or reduced costs [82]. Patient portals provide a technical basis for information exchange but still seem to be limited in their ability to really tailor content and feedback to patient input, which may limit their effectiveness in terms of skill development. Therefore, they may be enhanced by providing information and educational materials in a tailored way, for example, via intelligent algorithms that enable the tailoring according to the user’s age, eHealth literacy, and coping style. Patient empowerment could also be enhanced by adding tailored interventions, such as online cognitive behavioral therapy.

### Promising Developments and Research Priorities

Based on this study, we conclude that services that are able to elicit survivors´ most bothersome issues and provide them with guidance to improve these issues have great potential to empower them. Preferably, an active attempt to improve or reinforce skills (eg, their ability to cope with emotions or to deal with fatigue) is used. Services that are particularly promising in this regard are tailored ePRO systems such as SAM, ESRA-C, and OncoKompas [21,22,61]. A significant challenge will be defining thresholds (ie, cutoff values) for screening questionnaires of such services, and generating a decision tree with valid and acceptable interventions that align with existing care pathways. The stepped-care model, starting with self-management options and gradually progressing to professional help, is a promising approach. It is largely unknown what would be the optimal format for graphically presenting ePRO data to cancer survivors and their HCPs, but researchers are starting to look into this issue [83]. It may be useful to include question prompt sheets that enable patients to formulate their specific questions prior to a medical visit, based on their ePRO results. Most ePRO systems make use of validated quality-of-life and symptom measures. From an empowerment perspective, it may be useful to supplement these measures with questions that ask patients to identify their most relevant health problems and goals, as does the ESRA-C [61]. Future challenges in this regard will be to make these services aimed truly at the patients’ needs, effective in following up on the identified needs, and making these services attractive and easy to use for patients with varying levels of eHealth literacy and sociodemographic characteristics. An area that was not fully tapped into in this paper, but that also may be promising, is that of social media services such as Twitter and Facebook. There is, for example, recent literature indicating that Twitter may be valuable for patients to increase their knowledge [84] and their perceived support from the community [85].

Consequently, several research priorities can be pointed out. First, research needs to focus on how to best measure what survivors identify as key areas or goals that they want to work on regarding their health, and how these could be best met by existing health services, either on- or offline, in preferably a stepped-care manner. Second, research is needed to determine the optimal information provision to survivors given their informational coping styles. Third, it is important to determine the key skills that cancer survivors need, as well as the most effective ways to enhance these skills, possibly differentiated for different types of cancer. And finally, an important issue with health IT services is that there is a limited uptake. According to a large study in the United States (n=3959), overall use ranged from 3% to 78% for online diary keeping and health information seeking on the Internet, respectively. They also found that older persons, males, and those with a lower socioeconomic status were less likely to engage in a number of eHealth activities (odds ratio ~ 0.5) compared to their counterparts [86]. It may therefore be prudent to study ways to optimize the reach of such services in cancer survivors, for example, by using the Reach Effectiveness Adoption Implementation Maintenance (RE-AIM) framework that was developed to guide the widespread adoption and implementation of health interventions [87].

### Limitations and Strengths

First, regarding the integrative literature search on empowerment, we did not include or exclude articles based on quality criteria, which makes it impossible to objectively assign more weight to one article over another. Furthermore, it is an inherent limitation of this review that there is a limited number of studies specifically focusing on cancer patients. However, as findings with regard to patient empowerment in the larger body of literature on chronically diseased patient populations were also reflected in the more limited literature specifically focusing on patients with cancer, we can assume that the conceptualization of empowerment applies to the cancer setting as well. Second, the review of IT services may not include every IT service available at the time of this review. This is a rapidly developing area, and thus it is difficult to be comprehensive. Rather, we have provided an overview of some of the more widely publicized IT services that serve cancer survivors. Although there are no established methods for reviewing newly developed IT services, we have suggestions for future attempts. Reviews in this area may be strengthened by searching multiple databases (eg, PubMed and Scopus), having at least two persons evaluating the eligibility, extracting features/functionalities of the services, and relating the features/functionalities to predefined quality criteria when available. For example, Williams et al reviewed online decision aids and rated them on International Patient Decision Aid Standards (IPDAS) criteria [88]. Finally, our statements regarding the contribution of IT to patient empowerment have been based largely on hypothesized effectiveness, as the current evidence base in this area is quite small.

The major strength of this review is that it addresses both the literature on defining patient empowerment, and the literature on current IT services for cancer survivors and their possible contribution to enhancing empowerment. Another strength is the use of the integrated review method, which facilitates inclusion and integration of different sources and types of information (eg, theoretical and empirical manuscripts) in a single review [12].

### Conclusions

In this paper, we have identified the key attributes of the concept of patient empowerment for chronic disease patients including cancer survivors, and we have illustrated the ways in which IT services can contribute to enhancing empowerment of cancer survivors. We found that IT services were mainly related to knowledge provision (eg, about the patients’ medical conditions) and that active approaches for skill development were limited. Future challenges will be to make these services aimed truly at the patients’ needs, effective in following up on their identified needs, and making these services attractive and easy to use for patients with varying levels of eHealth literacy and sociodemographic characteristics.

## Appendix

Multimedia Appendix 1 Electronic PubMed searches.

Multimedia Appendix 2 Examples of IT services with potential for empowerment of cancer survivors.

## Abbreviations

A-CaRe: Alpe d’HuZes Cancer Rehabiliation
AR: being autonomous and respected
CBT: cognitive behavioral therapy
CCM: Chronic Care Model
CHES: Computer-based Health Evaluation System
CHESS: Comprehensive Health Enhancement Support System
EMR: electronic medical record
ePRO: electronic patient-reported outcome
ESRA-C: Electronic Self-Report Assessment for Cancer
HCP: health care provider
HK: having knowledge
HL7: Health Level Seven
ICT: information and communication technology
IDEA: Interactive Digital Education Aid
IPDAS: International Patient Decision Aid Standards
IT: information technology
IVF: in vitro fertilization
KWF: Dutch Cancer Society
o: no positive contribution to empowerment
PRISMA: Preferred Reporting Items for Systematic Reviews and Meta-Analyses
PRO: patient-reported outcome
PS: perceived support from community, family, and friends
RCT: randomized controlled trial
RE-AIM: Reach Effectiveness Adoption Implementation Maintenance
ROGY: Registrationsystem Oncological GYnecology
SAM: self-assessment and management
SCP: survivorship care plan
SHUTi: Sleep Healthy Using The Internet
SK: having skills
UMC: University Medical Center
+: weak positive contribution to empowerment
++: strong positive contribution to empowerment
