# Supplementary material for: Empowerment of Cancer Survivors Through Information Technology: An Integrative Review
Source: J Med Internet Res. 2015 Nov 27;17(11):e270. doi: 10.2196/jmir.4818 (PMC4704924; doi:10.2196/jmir.4818)
Supplement: Multimedia Appendix 1 [file jmir_v17i11e270_app1.pdf]

Multimedia Appendix 1. Electronic PubMed searches

Primary search on Patient Empowerment

((patient empowerment AND ((conceptual) OR (theory) OR (cancer))))
filter: English

Primary search on IT services for cancer survivors

(((("cancer patient" OR "cancer survivor" OR "cancer survivorship" OR “cancer”))))
AND ((("ict" OR "information and communication technology" OR "web-based" OR "internet"))
Filters: English; Reviews; publication date Jan 2010-Jan 2015
