# Supplementary material for: Empowerment of Cancer Survivors Through Information Technology: An Integrative Review
Source: J Med Internet Res. 2015 Nov 27;17(11):e270. doi: 10.2196/jmir.4818 (PMC4704924; doi:10.2196/jmir.4818)
Supplement: Multimedia Appendix 2 [file jmir_v17i11e270_app2.pdf]

Multimedia Appendix 2. Examples of IT services with potential for empowerment of cancer survivors

System name	Key features and user interaction aspects	Integration with electronic medical record (EMR)
**Patient Portals**		
MyChart/EPIC [48]	• View test results<br>• View upcoming & past appointments<br>• Fill out pre-visit questionnaires<br>• Schedule appointments<br>• View paperless statements & pay bills online<br>• Upload photos<br>• Connect to home devices<br>• Refill prescriptions<br>• Message securely with providers<br>• View a child's records and print growth charts<br>• Manage the care of elderly parents<br>• View education topics triggered by EHR data<br>• Get a chronic disease summary w/reminders	Yes
ChipSoft patient portal	• Access to parts of the medical record via secure portal<br>• View scheduled and past visits<br>• Provide and change registered patient personal data<br>• Fill out questionnaires prior to a visit by computer at home	Yes

	• presentation of questionnaire data in tables (for both patients and clincians)<br>• Clinicians can provide patient education for upcoming visits by selecting documents manuallly<br>• Access for the general practitioner (optional)<br>• Output (e.g advice) can tailored based on input data via specific predefined rules and is presented in the portal	
Digital IVF clinic (digitale IVF poli)	Access to:<br>• Planned visits<br>• Outcome of treatment<br>• Laboratory results<br>• Ultrasound results<br>• Letters to general practitioners<br>• Photos of the embryo<br>• Communication with the care team	No direct integration.
Medischegegevens.nl [47]	Access to:<br>• Medical data<br>• Letters to general practitioners<br>• Laboratory results<br>• Diagnostic test results<br>• Radiology results (Rontgen pictures)	No, medical data is extracted to patient portal surrounding.

	• Add own documents and allergies<br>• Make summary of health history<br>• Provide access to physicians of choice	
Care companion [46]	• Personal care trajectory depicted as a “metro trail” including all important phases of treatment with patient education linked to each “station”<br>• Library<br>• Information about diagnosis<br>• Overview of scheduled visits<br>• Personal notes<br>• Monitoring personal data (e.g. pain of QoL) and graphical presentation<br>• View and update personal data (such as name birth data etc.)	No direct integration
**Electronic patient reported outcome systems**		
CHES: Computer-based Health Evaluation System [50]	• Electronic filling out of quality of life questionnaires by patients on-site via computerized questionnaire (web based input at home is under construction).<br>• Graphical depiction of quality of life scores for different measurements<br>• Addition of reference values and flags for deviant scores<br>• Software can be modified to user requirements (e.g recording of chemotherapy details in oncological patients).<br>• Graphical overview currently only available for clinician	No, but database can be linked to EMR via HL7.
Electronic patient reported	• Possibility for patients to fill out questionnaire on symptoms and quality	Yes

outcome system of UMC Leiden, NL	of life at home<br>• Graphical overview of domain scores of quality of life for health professionals<br>A hospital employee manually :<br>• Sends out questionnaires to patients<br>• Notifies physicians of alarming scores<br>• Physicians are able to view quality of life profile in their own hospital information system	
Eir, a computer based decision support system [49]	• Patients complete symptom assessment at home on an tablet computer prior to consultation<br>• Data is transferred wireless to physician desktop<br>• Physician has an overview of symptoms and complaints prior to consultation<br>• Physician can add treatment and clinical data<br>• Clinical decision support is provided by the system based on input data and is based on international guidelines	unknown
Electronic Patient reported outcomes from cancer survivors (ePOCS) [45].	The two key components of the ePOCS system are QTool, a custom-designed Web-based password-protected questionnaire administration and management system, and the Tracker, a custom-designed database for monitoring patients' QTool activity and generating study correspondence (eg, invitations to complete questionnaires, reminders), which is housed on a secure registry server.<br>*QTool functionalities*<br>• Any PROM can be entered and displayed in various formats (e.g., one or multiple items per screen, tick-box or drop-down menu response	No, data are merged together with EMR data and then sent to the national cancer repository.

options).

- Individual PROMs questions can be set to display (or not), dependent on responses to other questions, and to require an answer (or not).
- Any combination of PROMs can be assigned to any size patient group, and different groups can complete different PROMs packages simultaneously.
- PROMs can be set to be accessible to patients at any specific time-points from their diagnosis date, and to be available for any window of time (e.g., PROMs to be completed 6 months post diagnosis, within 4 weeks, would be accessible from July–August for a patient diagnosed in January, and from October–November for a patient diagnosed in April).
- Entering and setting PROMs is menu-driven and does not require specialist skills.

Tracker functionalities

- On a daily basis, all due correspondence is automatically calculated for all patients in the system, for every data collection time point (e.g., invitations to complete PROMs, reminder notices, thank you acknowledgements).
- Appropriate pre-programmed communications are automatically selected and populated with the relevant patient's details (e.g., name, address, QTool ID + password).
- Prepared communications are generated ready to send as Emails, or to print out as letters, dependent on whether a patient provides an Email address or not.

	• Any communication can be recorded as unsent (e.g., if a patient is unwell and requests communications are stopped).<br>• All sent and unsent communications associated with a patient can be instantly viewed in a table.<br>• Notice is provided of patients near-due to complete further PROMs, along with a printable details form (e.g., name, address, NHS number), so administrators may check patient status (e.g., via GP), to ensure it is appropriate to send a PROMs invitation.	
Electronic patient self-assessment (SAM) [51]	• Patients get invitation by email to fill out validated questionnaires online at preset intervals post-surgery (mainly developed for prostate cancer patients undergoing surgery).<br>• Results of questionnaires are analyzed to provide patients with real-time and, online information about their progress and to provide them with tailored and standardized medical advice.<br>• Data could be electronically transferred to the health care provider.	No, but data could be transferred to the clinician electronically
OncoKompas [52]	• A personal website that provides a profile of health status (depicted as a compass) is provide based on user input.<br>• Per health status domain it is shown if a person is on track (shown as green, orange or red signs).<br>• When orange or red, then tailored advises (via stepped care order progressing from self-help to suggestions for professional medical intervention.	No
**Electronic survivorship care (plan) support**		
Livestrong Care Plan [53]	A personal survivorship care plan (SCP) is generated based on patient input. The SCP contains:	No[10]

	• Records of medical history.<br>• Specific information about cancer diagnosis and treatment.<br>• Information about possible late effects and signs of a recurrence or new cancer.<br>• A schedule for follow-up health care including screening tests.<br>• Tips on cancer prevention and suggestions for maintaining a healthy lifestyle.<br>• How to find quality health care and other support services.	
Survivorship Care Plan builder [54]		No, but it is possible to export patient data from from C/NExT registry software to the Survivorship Care Plan builder
ROGY Care [55]	• A paper based personal survivorship care plan (SCP) is generated based on a registration system for gynecological cancer. The SCP provides tailored information based on personal patient and disease data (e.g., name patient, date of birth, type of cancer, cancer stage, treatment received, providers involved).<br>• Detailed information is provided on the diagnosis, treatment, possible short term and long-term effects of the disease and the treatment and aftercare.	No, but is linked to a separate regional cancer registry system for gynecological tumors.

- Recurrences, toxicities or other specialists involved in the patient’s care are registered in ROGY and automatically updated in the personal SCP.
